# Intestinal schistosomiasis in Uganda at high altitude (>1400 m): malacological and epidemiological surveys on Mount Elgon and in Fort Portal crater lakes reveal extra preventive chemotherapy needs

**DOI:** 10.1186/s40249-017-0248-8

**Published:** 2017-02-06

**Authors:** Michelle C. Stanton, Moses Adriko, Moses Arinaitwe, Alison Howell, Juliet Davies, Gillian Allison, E. James LaCourse, Edridah Muheki, Narcis B. Kabatereine, J. Russell Stothard

**Affiliations:** 1 Department of Parasitology, Liverpool School of Tropical Medicine, Pembroke Place, Liverpool, L3 5QA UK; 2 Vector Control Division, Ministry of Health, Kampala, P.O. Box 1661, Uganda; 3 Schistosomiasis Control Initiative, Imperial College London, London, W2 1PG UK

**Keywords:** School children, *Schistosoma mansoni*, Kato-Katz, CCA, SEA-ELISA, *Biomphalaria*

## Abstract

**Background:**

Intestinal schistosomiasis is of public health importance in Uganda but communities living above 1400 m are not targeted for control as natural transmission is thought unlikely. To assess altitudinal boundaries and at-risk populations, conjoint malacological and epidemiological surveys were undertaken on Mount Elgon (1139 m–3937 m), in Fort Portal crater lakes and in the Rwenzori Mountains (1123 m–4050 m).

**Methods:**

Seventy freshwater habitats [Mount Elgon (37), Fort Portal crater lakes (23), Rwenzori Mountains (8) and Lake Albert (2)] were inspected for *Biomphalaria* species. Water temperature, pH and conductivity were recorded. A parasitological examination of 756 schoolchildren [Mount Elgon (300), Fort Portal crater lakes (456)] by faecal microscopy of duplicate Kato-Katz smears from two consecutive stool samples was bolstered by antigen (urine-CCA dipstick) and antibody (SEA-ELISA) diagnostic assays.

**Results:**

*Biomphalaria* spp. was found up to 1951 m on Mount Elgon and 1567 m in the Fort Portal crater lakes. Although no snail from Mount Elgon shed cercariae, molecular analysis judged 7.1% of snails sampled at altitudes above 1400 m as having DNA of *Schistosoma mansoni*; in Fort Portal crater lakes three snails shed schistosome cercariae. Prevalence of intestinal schistosomiasis as measured in schoolchildren by Kato-Katz (Mount Elgon = 5.3% v. Fort Portal crater lakes = 10.7%), CCA urine-dipsticks (18.3% v. 34.4%) and SEA-ELISA (42.3% v. 63.7%) showed negative associations with increasing altitude with some evidence of infection up to 2000 m.

**Conclusions:**

Contrary to expectations, these surveys clearly show that natural transmission of intestinal schistosomiasis occurs above 1400 m, possibly extending up to 2000 m. Using spatial epidemiological predictions, this now places some extra six million people at-risk, denoting an expansion of preventive chemotherapy needs in Uganda.

**Electronic supplementary material:**

The online version of this article (doi:10.1186/s40249-017-0248-8) contains supplementary material, which is available to authorized users.

## Multilingual abstracts

Please see Additional file 1 for translations of the abstract into the six official working languages of the United Nations.

## Background

Schistosomiasis is of considerable public health importance in sub-Saharan Africa [1]. Across the continent there are several national control programmes (NCPs) operating at various levels of scale-up, as guided by expectations set out within the WHO 2012–2020 Roadmap for neglected tropical diseases [2, 3]. In Uganda, since 2003 there has been an active NCP primarily concerned with the delivery of praziquantel to school-aged children [4, 5]. Whilst each form of schistosomiasis can be found in Uganda, intestinal schistosomiasis, caused by *Schistosoma mansoni* (and transmitted by *Biomphalaria* spp.), is most common and widespread [6–8]. Just under 20 million people in 73 districts are estimated to be at-risk of infection [2]. The disease-endemic zone is most obvious within shoreline habitats of the Great Lakes and River Nile as well as in its vicinities nearby [3]. Outside of these however, large-scale ecological and epidemiological predictions have broadly set aside such areas as being unlikely to sustain natural transmission, for example, too cold (altitude > 1400 m) or too arid (annual rainfall < 90 cm) [9–12].

Alongside general surveillance and monitoring activities within the Ugandan NCP, an associated programme of operational and cross-country collaborative research has been undertaken [13, 14]. This has explored the dynamics of environmental transmission in East Africa, especially in areas poorly sampled previously [15, 16]. Schistosome-snail ecology has been addressed at either macro- and micro-epidemiological levels; many heterogeneities in biotic and (or) non-biotic factors have come to light [17–27]. In addition, more sensitive diagnostics tools have been introduced which go beyond routine parasitological sampling which has in turn increased abilities to better detect and differentiate schistosome infection(s) either in people [28, 29] or in snails [30–32]. Furthermore, with advances in geographical information systems and more affordable digital cartography [27, 33–36], new opportunities arise to review and refine earlier eco-epidemiological predictions. This includes targeted epidemiological surveys to ‘ground-truth’ assertions now better armed with new diagnostics.

This paper is an attempt to determine the risk of intestinal schistosomiasis transmission at higher altitudes in Uganda using data from two prospective malacological and epidemiological surveys conducted on Mount Elgon and in Fort Portal crater lakes. The surveys also make reference to Lake Albert and Rwenzori Mountains, assessing if natural transmission of intestinal schistosomiasis occurs at altitudes near to or exceeding 1400 m.

## Methods

### Malacological surveys and laboratory investigations

At each freshwater habitat surveyed, global position system (GPS) coordinates, altitude and location photographs were taken with an Oregon 650 receiver (Garmin, Olathe, Kansas, USA). Water temperature (°C), pH and conductivity (μS) were recorded with a HI-98129 Pocket EC/TDS and pH Tester (Hanna Instruments Ltd, Leighton Buzzard, Bedfordshire, UK). Two snail collectors searched for *Biomphalaria* spp. by hand and with metal scoops, for over 20 min at each site. All collected snails were counted then transferred into plastic cups containing mineral water, exposed to light for two hours, then checked for shedding cercariae under the dissecting microscope. Thirty seven sites (1139 m–3937 m) were surveyed in June 2011 in the Mount Elgon area, to include areas located close to sampled schools (see below). Within the Fort Portal crater lakes, 23 sites (1123 m–1567 m) were surveyed in June 2012, including a selection of those previously visited by Rubaihayo *et al*. in 2006 [37], with sampling taking place in areas of the lakes known to be used by local communities for fishing and domestic activities. Eight further higher altitude sites were surveyed by foot in the Rwenzori Mountains (1620 m–4050 m), plus an additional two by car on the southern shore of Lake Albert (616 m and 624 m) as low altitude reference.

*Biomphalaria* spp. collected from Mount Elgon was placed in absolute ethanol, transferred to the UK and genomic DNA was extracted according to standard protocols. A total of 118 snails (33 from < 1400 m and 85 from > 1400 m) were then screened by polymerase chain reaction (PCR) for schistosome DNA following protocols described by Kane *et al*. [32] using schistosome–specific primers, RAKqIGSF (5’ AAA GTC GGA AAA ATG AAA 3’) and RAKqIGSR (5’ TAT GAA TGA AAT CGG TTA 3’) for a sub-region of the nuclear ribosomal intergenic spacer [32]. Amplicons were separated by 1.5% agarose gel electrophoresis and stained with ethidium bromide. A snail was judged to be infected with *S. mansoni* if a 300 base pairs fragment that could also be digested with the restriction enzyme *Acc*1 was observed [32].

### Epidemiological surveys and diagnostic assays

The GPS coordinates and elevation of each sampled school were recorded. Within the Mount Elgon area, six universal primary education (UPE) schools were included: 3 at higher altitudes (1856 m–2072 m) and three at lower altitudes (1150 m–1268 m). Schools were sampled randomly within two elevation strata (<1500 m and >1500 m). Within each school, 50 children were randomly selected (25 boys, 25 girls) from classes Primary 5 – Primary 7 (age range of 10–13 years). Within the Fort Portal Crater Lakes area, 14 UPE schools (1165 m–1526 m) were selected, six of which had been surveyed previously by Rubaihayo *et al*. in 2006 [37]. The additional schools were selected purposively such that they covered a range of altitudes and were close to a crater lake. An additional school was sampled close to the southern shores of Lake Albert (621 m), an area where transmission is known to be high, as a comparison. Within each school, 30 children were examined. No school was sampled in the Rwenzori Mountains as the survey took place within the Rwenzori National Park.

Specimen collection and diagnostic testing took place over a two day period with methods described previously [38]. Urine, stool and finger-prick blood samples were taken on the first day, with an additional stool sample obtained on the second day. Faecal microscopy involved inspection of duplicate thick Kato-Katz smears from each faecal sample with egg counting at ×100 magnification. A urine-CCA dipstick test (Rapid Medical Diagnostics, Pretoria, South Africa) was used to detect intestinal schistosomiasis and 3 μl of harvested sera was used for detection of antibodies against soluble egg antigen (SEA-ELISA) with a commercially available kit (IVD Inc.; Carlsbad, USA). All sampled children were treated on site with praziquantel (40 mg/kg, Cipla, Mumbai, India) and albendazole (400 mg, GSK, Hertford, Hertfordshire, UK) irrespective of infection status.

### Spatial and statistical analyses

The relationship between altitude and the survey results was explored through the production of maps and scatter plots using the geographical information software QGIS (version 2.18.1) [39] and the statistical software R (version 3.3.1) [40]. In addition to mapping the geographical coordinates of the survey sites, elevation data at a 90 m resolution was obtained from NASA’s Shuttle Radar Topography Mission (SRTM) [41] and displayed using elevation bands of less than 1000 m, 1000–1200 m, 1200–1400 m, 1400–1600 m, 1600–1800 m, 1800–2000 m and greater than 2000 m for the whole of Uganda. With regards to the malacological survey data, in addition to plotting elevation against number of *Biomphalaria* spp. caught at each site, plots of water temperature (°C), pH and conductivity against elevation were produced in order to explore further this relationship.

Plots of school prevalence using all three diagnostic methods were produced and a relationship between the pooled prevalence results using each of the three diagnostic methods and elevation was assessed by fitting a logistic regression model to the data. The goodness of fit of these models was assessed by calculating the deviance statistic [42]. In order to explore the currently available information on high altitude schistosomiasis transmission excluding the surveys presented in this paper, georeferenced schistosomiasis prevalence data obtained primarily using Kato-Katz were downloaded from the Global Atlas of Helminth Infections (GAHI) website [43]. This database is a collation of prevalence data from multiple sources including the published literature and the national NTD control programme [33, 44, 45]. The elevation of each georeferenced point was extracted from the 90 m resolution SRTM data, and summaries of surveys, including the number undertaken and the reported prevalence ranges by elevation bands, were reported.

The altitude of each georeferenced point was extracted from the 90 m resolution SRTM data, and summaries of surveys, including the number undertaken and the reported prevalence ranges by elevation bands, were reported. Finally, using 100 m gridded population data for 2015 obtained from WorldPop (http://www.worldpop.org.uk/) the number of people living within elevation bands of less than 1400 m, 1400–2000 m and greater than 2000 m was calculated in order to obtain a crude estimate of the number of people at-risk.

## Results

### Malacological findings

**Fig. 1:**
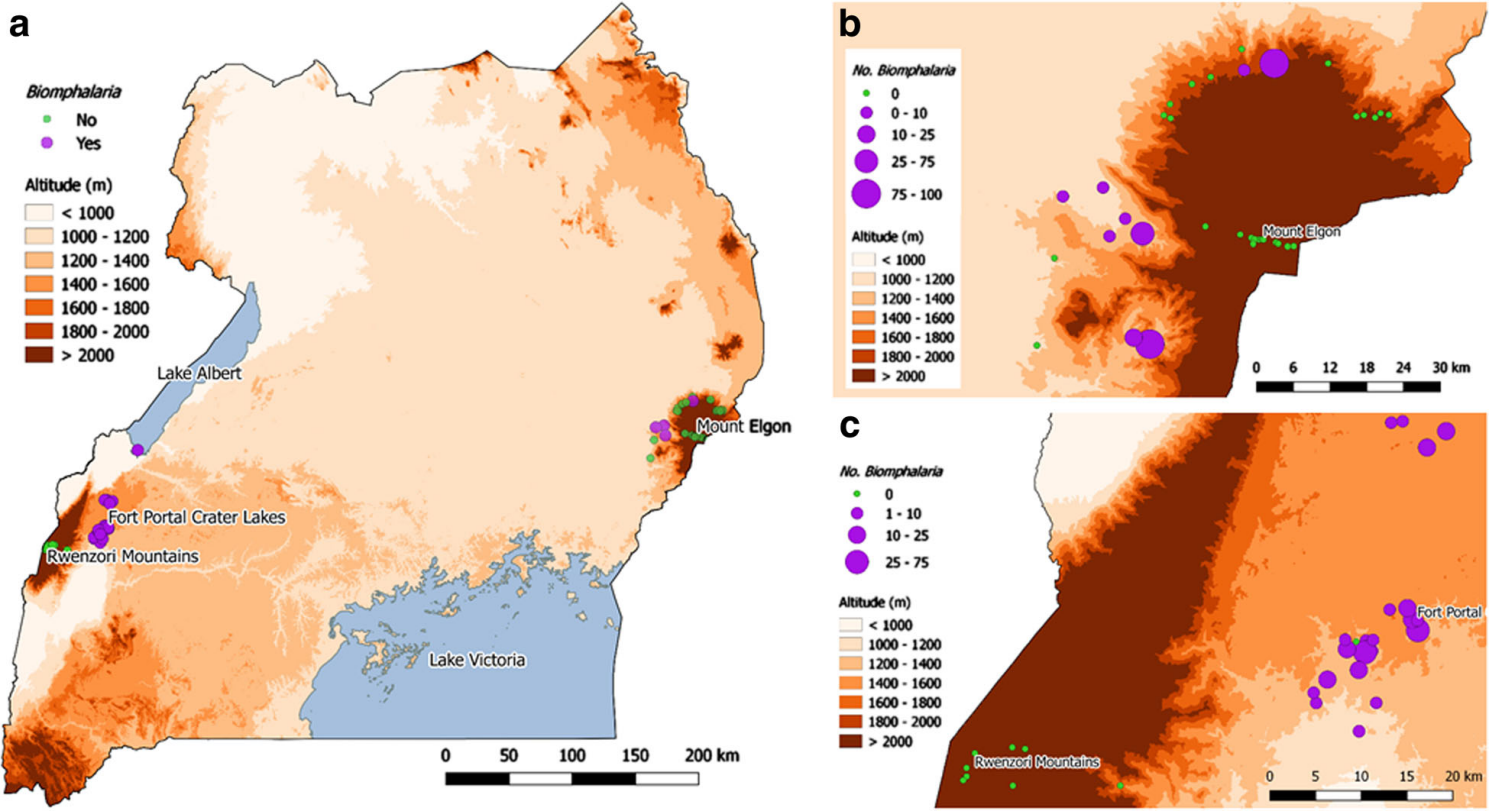
Outline maps of Uganda showing altitude and malacological sampling points. **a** Country overview of Mount Elgon (East) and Fort Portal crater lakes and Rwenzori Mountains (West); sample sites where *Biomphalaria* spp. were found are indicted *purple* whereas *green* denotes no *Biomphalaria* spp. encountered. **b** Mount Elgon area **c** Fort Portal Crater Lakes and Rwenzori Mountains and area

**Fig. 2:**
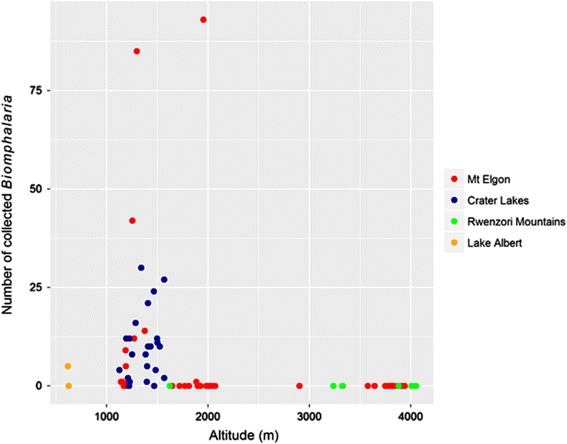
Scatterplot of number of *Biomphalaria* spp. collected at each site against altitude

**Fig. 3:**
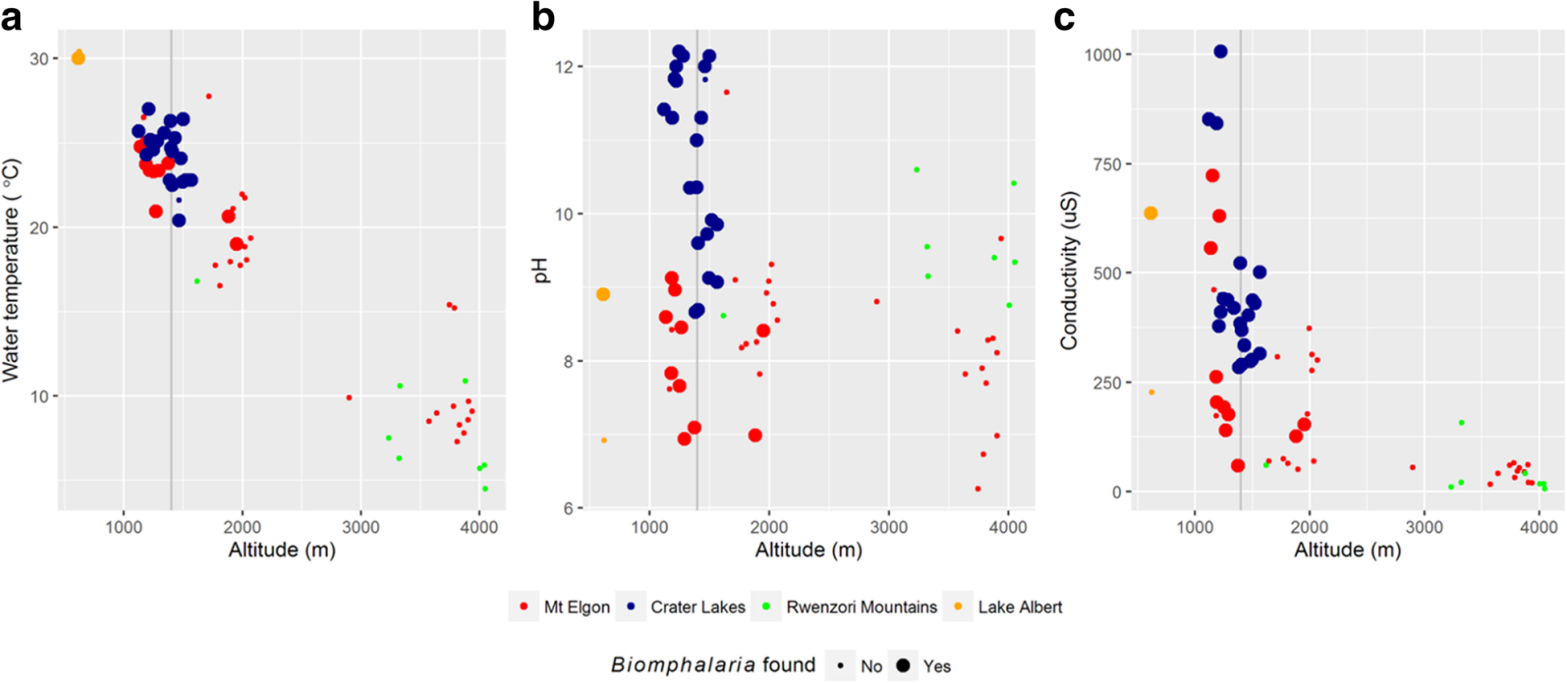
Scatterplot of water temperature (**a**), pH (**b**) and conductivity (**c**) at each snail site (Mount Elgon = *red*, Crater Lakes = *blue*, Rwenzoru Mountains = *green*, Lake Albert = *orange*) against altitude for all survey areas. *Large circles* represent sites were at least one *Biomphalaria* spp. was found

Figure 1 presents an overview of the locations of the snail sampling sites across Uganda, with all data in Additional file 2: Table S1. Within the Mount Elgon area, *Biomphalaria* spp. were found at 30% (11/37) of sampled sites at altitudes as high as 1951 m whereas in the Fort Portal crater lakes were found 92% (21/23) of sampled sites, at a maximum altitude of 1567 m. No *Biomphalaria* spp. were found in the Rwenzori Mountains. *Biomphalaria* spp. were found in one of the two low altitude sites on Lake Albert. Scatter plots of number of *Biomphalaria* spp. against altitude (Fig. 2) indicated that no *Biomphalaria* spp. were found above 2000 m. There was no discernible pattern in the number of snails and altitude below this value. Plots of water temperature, pH and conductivity against altitude, indicate that whilst there appears to be a negative linear relationship between temperature and altitude, see Fig. 3, the water in Mount Elgon has a lower pH (median = 8.3) than that of Fort Portal crater lakes area (median = 11.3) and the Rwenzori Mountains (median = 9.5). There is no clear trend between pH and altitude or *Biomphalaria* spp.. A negative association of conductivity with increasing altitude was observed, with the lowest values (<100 μS) being found at sites at very high altitudes (>3000 m).

A total of 264 *Biomphalaria* spp. collected from Mount Elgon were observed for shedding cercariae under natural conditions on at least one occasion. While non-human cercariae were seen, no snail shed schistosome cercariae. Upon PCR analysis conducted in the UK, 16 snails from a total of 33 screened from below 1400 m and six snails from a total of 85 screened from above 1400 m were judged infected with *S. mansoni* with a prevalence of 48.5 and 7.1%, respectively. In total, 235 *Biomphalaria* spp. collected from Fort Portal crater lakes were observed for shedding cercariae, with three snails from Nyinambuga and Lyantonde, two independent crater lakes, patently shedding schistosome cercariae.

### Epidemiological findings

**Fig. 4:**
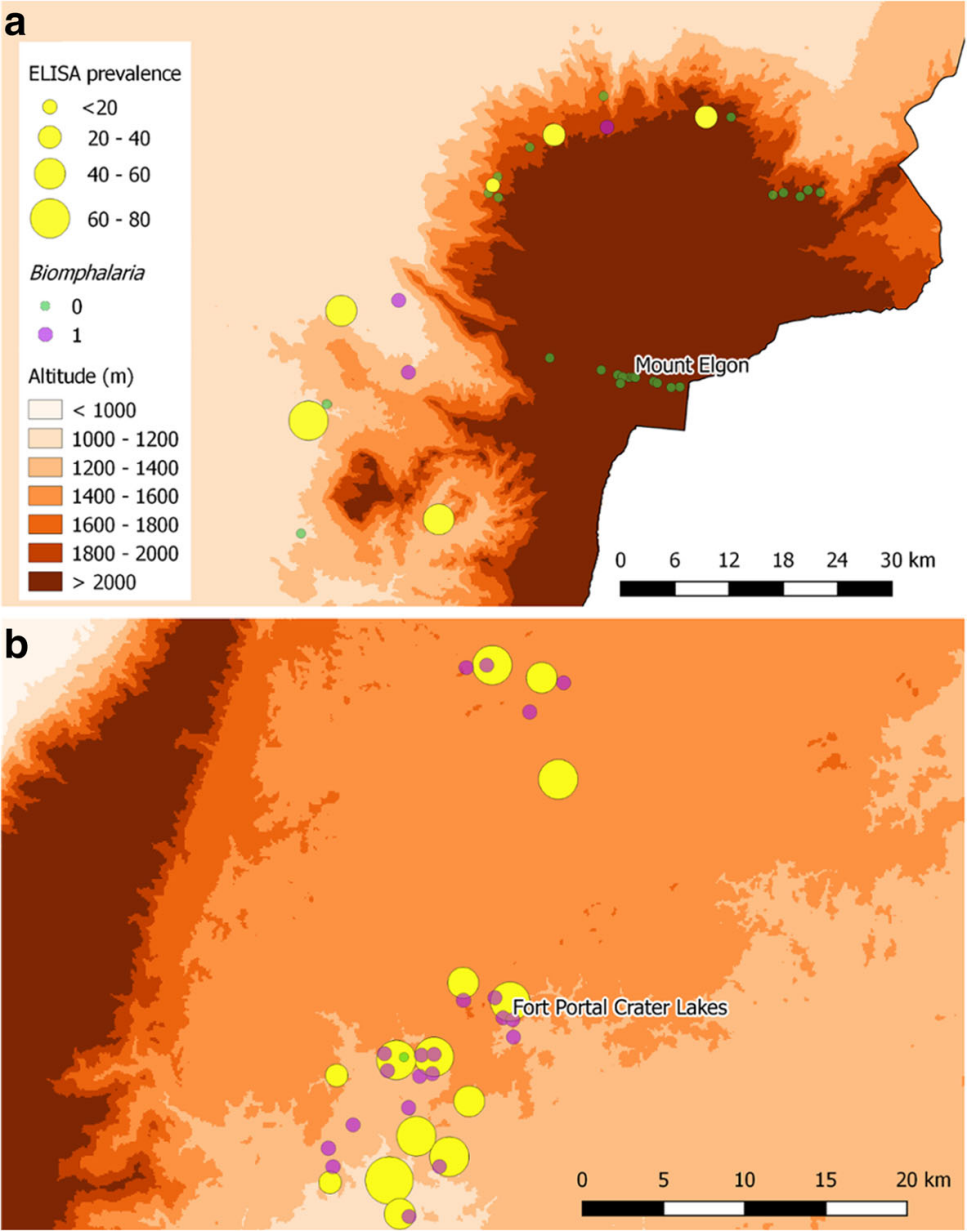
Schematic maps of prevalence of intestinal schistosomiasis at each surveyed schools according to SEA-ELISA depicted as *yellow* coloured circles in the Mount Elgon (**a**) and Fort Portal crater lakes (**b**) areas. Snail sampling sites are also depicted as *purple* (*Biomphalaria* present) and *green* (*Biomphalaria* spp. absent) coloured circles

**Fig. 5:**
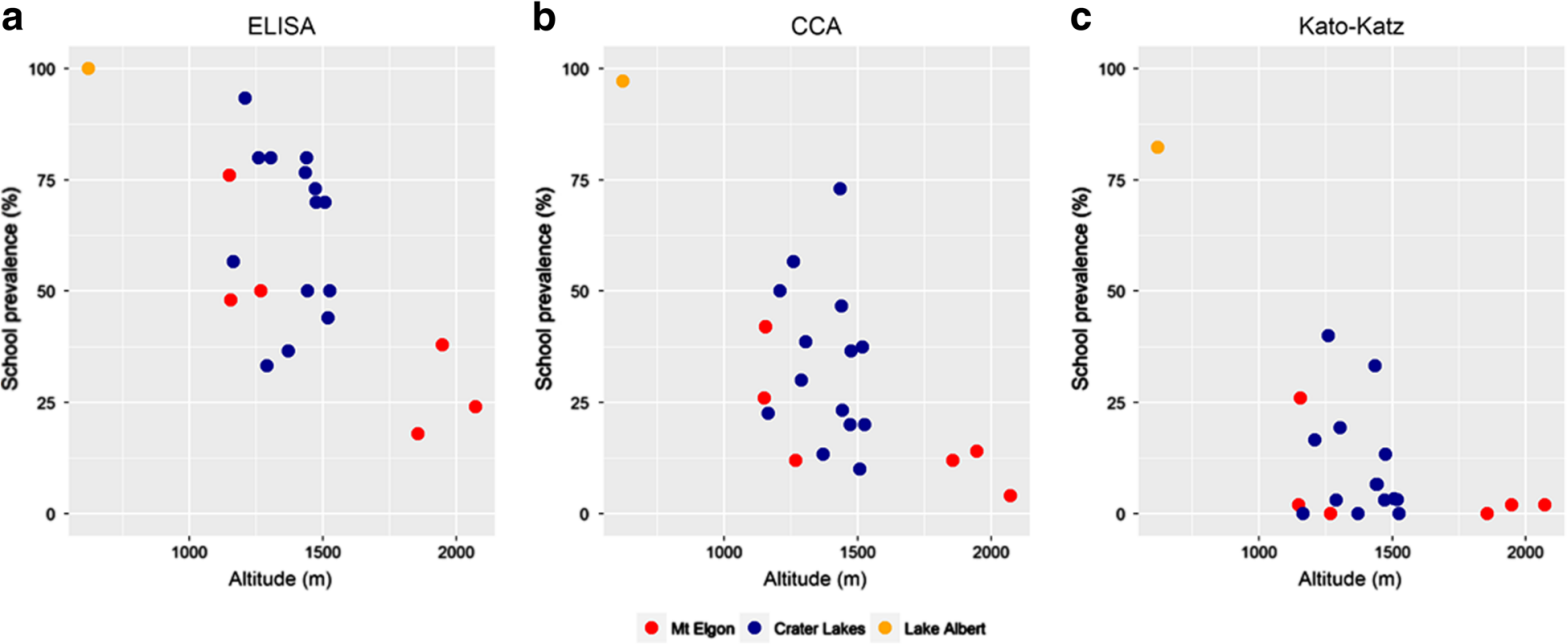
Scatter plots of prevalence of intestinal schistosomiasis by school according to SEA-ELISA prevalence (**a**), CCA urine-dipstick (**b**) and Kato-Katz (**c**) show a negative relationship with increasing altitude

Table 1 presents the overall prevalence of *S. mansoni* within schools sampled from Mount Elgon, Fort Portal crater lakes and Lake Albert as measured by Kato-Katz, CCA and SEA-ELISA. Prevalence by SEA-ELISA testing in the Mount Elgon and Fort Portal crater lakes areas are shown in Fig. 4, with Fig. 5 depicting school-level prevalence against altitude for each of the three diagnostic methods. Prevalence as obtained with each diagnostic decreases with increasing altitude (SEA-ELISA: Lake Albert = 100%, Fort Portal crater lakes = 60.4%, Mount Elgon = 42.3%). Individual school data can be found in Additional file 3: Table S2. According to SEA-ELISA testing there is strong evidence of infection at higher altitudes, such that a prevalence of 26.7% (40/150) was detected in the three most elevated schools (1856–2072 m), falling to 10.0% (15/150) and 1.3% (2/150) using CCA and Kato-Katz, respectively. Of note, is that these schools are within reasonable proximity (approximately 10 km) to a location where numerous *Biomphalaria* spp. were found, Fig. 4.

**Table 1:** Summary of school-level schistosomiasis prevalence by area and diagnostic method

	Elevation	*N*	SEA-ELISA		CCA		Kato-Katz	
			Prevalence (%)	95% *CI*	Prevalence (%)	95% *CI*	Prevalence (%)	95% *CI*
Mount Elgon	1150–2072	300	42.3	36.7–48.2	18.3	14.2–23.3	5.3	3.2–8.7
Crater Lakes	1165–1526	420	63.7	58.9–68.3	34.4	29.9–39.1	10.7	8.0–14.1
Lake Albert	621	30	100.0	87.4–100.0	97.1	82.9–99.8	70.1	52.3–84.3

The logistic regression model fitted to the prevalence data obtained using each of the three diagnostic methods, with elevation as the only risk factor, are presented in Table 2. The resulting odds ratios were 0.9977 (95% *CI*: 0.9947–0.9966), 0.9974 (95% *CI*: 0.9967–0.9980) and 0.9957 (95% *CI*: 0.9947–0.9966) for SEA-ELISA, CCA and Kato-Katz respectively. Thus, at the 1400 m threshold the fitted models predict prevalence values of 59.0% (95% *CI*: 55.2%–62.7%), 29.8% (95% *CI*: 26.4–33.4%), 7.8% (95% *CI*: 5.9–10.2%) for ELISA, CCA and Kato-Katz, respectively. It should be noted however that these models had a poor goodness of fit (*P* < 0.05).

**Table 2:** Logistic regression results for school-level schistosomiasis prevalence with elevation as a risk factor by each diagnostic method

		SEA-ELISA		CCA		Kato-Katz	
		Coefficient	95% *CI*	Coefficient	95% *CI*	Coefficient	95% *CI*
Log *OR*	Intercept	3.49	2.74, 4.28	2.74	1.92, 3.61	3.52	2.38, 4.77
	Elevation	−0.0022	−0.0028, −0.0017	−0.0026	−0.0032, −0.0020	−0.0043	−0.0053, −0.0034
*OR*	Intercept	32.81	15.43, 72.21	15.44	6.81, 36.88	33.75	10.77, 117.38
	Elevation	0.9978	0.9972, 0.9983	0.9974	0.9967, 0.9980	0.9957	0.9947, 0.9966

Of the 671 geolocated survey sites in the GAHI database (excluding the seven surveys whose coordinates were outside of the country boundary), 93.7% (629/671) were below an altitude of 1400 m. Of the remaining 42 surveys, 17 were within the 1400–1600 m range, five were within 1600 m–1800 m and 14 were within 1800 m–2000 m. The majority of surveys (94.3%, 633/671) used single Kato-Katz faecal smears to estimate prevalence of *S. mansoni* with egg-patent prevalence ranging from 0.0 to 57.0% (1400 m–1600 m), 0.0–1.7% (1600–1800 m) and 0.0%–3.4% (1800–2000 m). No cases were found at any sites at an altitude greater than 2000 m.

### Estimating the at-risk population

Using the gridded population estimates from WorldPop (100 m) and SRTM elevation (90 m), of the 39.2 million people living in Uganda, 82.8% (32.5 million) of people live at altitudes below 1400 m. Of the remainder, 15.4% (6 M) live within an altitude range of 1400 m–2000 m and the remaining 1.8% (0.7 million) live above 2000 m.

## Discussion

The analysis presented in this paper demonstrates the potential for intestinal schistosomiasis transmission at altitudes greater than 1400 m. This was clearly demonstrated both by the presence of disease and by the presence of intermediate snail hosts in Mount Elgon and Fort Portal crater lakes, respectively. The prevalence of disease at these higher altitudes should not be overlooked, and as such should be considered for inclusion in the national control programme.

For natural transmission of intestinal schistosomiasis to occur, several critical aspects within the lifecycle of *S. mansoni* need to be fulfilled [22, 34, 46, 47]. Foremost perhaps, is the presence of permissive populations of *Biomphalaria* spp., alongside sufficient environmental opportunity for larval stages of the schistosome to encounter and successfully develop within both definitive and intermediate hosts. As might be expected, there is an optimal range of temperatures operating across these processes both in time and space which may facilitate or, where thermal boundaries are exceeded by being too cold or too hot, stall natural transmission [22]. Hence there is ample reason and often sufficient ecological evidence to attempt to predict where transmission is or is not possible. There are, however, well-known conceptual problems broadly grouped as issues of geographical scale [27, 35, 36, 48]. In terms of public health, the implication of this can be profound for it may under-estimate or over-estimate the preventive chemotherapy needs of people living within areas predicted or proven to be at-risk [9, 16, 49]. Where resources permit, it is therefore sensible to have iterative cycles that progressively refine any prediction(s) such that subsequent validations are undertaken to ‘ground-truth’ any newly envisaged scenario.

The first formal attempt to describe the distribution of *Biomphalaria* spp. in Uganda was by Georg Mandahl-Barth in his 1954 monograph entitled “*The freshwater mollusks of Uganda and adjacent territories*” [50]. His attention focused most on *Biomphalaria* spp. from the great lakes and in lowland areas but mention was made of the crater lake fauna south of Fort Portal where he considered *Biomphalaria adowensis adowensis* (Bourguignat, 1879) to occur. Realising that his knowledge of the distribution and ecology of many species was scant, he provided only a broad overview but observed that *Biomphalaria rṻppellii* (Dunker, 1848) was found in highland areas in the South-West of the country within Lakes Mutanda (1800 m) and Bunyonyi (1962 m) [50]. Since then many of the older species names of *Biomphalaria* have been synonymised as the genus has been downwardly revised to represent some 12 species in total [51]. Most importantly all currently named species have some natural or experimental compatibility with *S. mansoni* hence the presence of *Biomphalaria* spp. alone is sufficient to raise suspicion of local transmission potential [17, 51]. Clearly from the information provided here, *Biomphalaria* spp. has been proven to be found at altitudes very close to 2000 m on Mount Elgon and with reference to Lake Bunyonyi was first reported in the 1930s [50] and is present today.

Even with the introduction of molecular DNA typing methods for species delineation, it remains difficult to describe and record precisely the distribution of each species of *Biomphalaria* in Uganda [52]. A good example is the status of populations in Lake Victoria although there have been successful attempts to map, describe and predict general distributions throughout the lake [15, 24–26, 38]. In terms of assessing transmission, recourse to a combination of traditional methods to inspect snails for evidence of infection, by cercarial shedding for example, alongside new DNA assays to detect schistosomes in snails is a powerful way to assess transmission in nature [32, 38]. With the observation of schistosome cercariae, it is apparent that active transmission was caught in action and was occurring during the survey of the Fort Portal crater lakes. It is perhaps unsurprising that the previous survey of Rubaihayo *et al*. found egg-patent prevalence of 27.8% around the crater lakes within the altitudinal range of 1487 m–1682 m [37]. There are also several recent reports of travellers contracting intestinal schistosomiasis locally from these crater lakes [53]. While shedding cercariae were not observed in the Mount Elgon survey, 7.1% of examined snails did have evidence of schistosome DNA which at the very least, demonstrates either recent encounters with schistosome miracidia or that snails were incubating sporocysts within the pre-patent period before cercariogenesis [54]. With hindsight our malacological surveys should have inspected a sub-sample of snails by crushing to visualise the presence of sporocyts and bolster this with recourse to real-time PCR approaches which can better quantify levels of schistosome DNA than traditional PCR methods using gel electrophoresis. With better quantification of DNA it should be possible to set a detection threshold which if exceeded, differentiates miracidial-contamination from cercarial-emergence events.

With the introduction of more sensitive diagnostics for intestinal schistosomiasis in people which clearly evidence the limitations of Kato-Katz, it is not surprising that a greater number of infections were encountered [29]. Figs. 4 and 5 demonstrate that while egg-patent prevalence was less than 5.0%, using SEA-ELISA or CCA the prevalence would fall somewhere between 5.0 and 37.5% in UPE schools above 1400 m. It is therefore safe to assume that rather than schistosomiasis being absent, it only appears absent due to the use of insensitive diagnostic tests. Table 2 shows that while there is a negative association with altitude there is a significant amount of infection between 1400 and 1600 m, with a likely absolute boundary exceeding 2000 m, although it should be noted however that these logistic regression models had a poor goodness of fit (*P* < 0.05) possibly due to the lack of additional important risk factors in the model or the small sample size. More data are therefore needed to explore this finding further. As an example, a prior survey for intestinal schistosomiasis at Hamukaaka (also known as Amasiko) village was undertaken on the shoreline of Lake Buynonyi (~1960 m) in 2006 and tested both pre-school children (*n* = 26) and their mothers (*n* = 29) by finger prick SEA-ELISA, urine-CCA dipsticks and stool concentration methods. Although *Biomphalaria* spp. could be found locally, there was no evidence of schistosome infection in children or in adults [55].

For those people living above 1400 m who are egg-negative by Kato-Katz but are positive by SEA-ELISA or urine-CCA test should have access to praziquantel treatment. Moreover, the significance of ‘asymptomatic’ schistosomiasis has been debated, concluding that there is a tangible benefit to treating those who have any evidence of infection [56]. Using available data on population density it is safe to assume that some 6.7 million people live within inhabited areas above 1400 m and that a sizeable fraction, perhaps a quarter to a half, will likely have intestinal schistosomiasis. However, these people have not yet been reached with treatment. Rather than set aside these areas for control, there is now an imperative for expanded access to preventive chemotherapy to be undertaken. This is especially true if control ultimately aims to eliminate morbidity and transmission, otherwise intestinal schistosomiasis could continue on within such high altitude refugia.

## Conclusions

These conjoint parasitological and malacological surveys undertaken on Mount Elgon and in the Fort Portal Crater Lakes clearly show that natural transmission of *S. mansoni* occurs at altitudes above 1400 m, with a putative upper boundary of 2000 m, and reveals additional preventive chemotherapy needs. In future, collected snails should also be examined for the presence of developing sporocysts by microscopy as well as implement real-time PCR techniques to better quantify levels of schistosome DNA snails with pre-patent infection(s). In these highlands, there is an appreciable burden of intestinal schistosomiasis in school children attending UPE schools. Using spatial epidemiological predictions, this now places some extra six million Ugandans at-risk of disease and calls for a much needed expansion of preventive chemotherapy by the Ugandan NCP in these highland areas. This will not only provide more equitable distribution of praziquantel treatment but also prevent schistosomes from making transmission refugia at higher altitude.

## Additional files

Additional file 1: Multilingual abstracts in the six official working languages of the United Nations. (PDF 809 kb)
Additional file 2: Table S1. Malacological data. (XLSX 16 kb)
Additional file 3: Table S2. Epidemiological data. (CSV 1 kb)

## Abbreviations

CCA: Circulating cathodic antigen
GAHI: Global atlas of helminth infections
NCP: National control programme
PCR: Polymerase chain reaction
SEA-ELISA: Soluble egg antigen-enzyme linked immunosorbent assay
SRTM: Shuttle radar topography mission
UPE: Universal primary education
