# Supplementary material for: Intestinal schistosomiasis in Uganda at high altitude (>1400 m): malacological and epidemiological surveys on Mount Elgon and in Fort Portal crater lakes reveal extra preventive chemotherapy needs
Source: Infect Dis Poverty. 2017 Feb 6;6:34. doi: 10.1186/s40249-017-0248-8 (PMC5292801; doi:10.1186/s40249-017-0248-8)
Supplement: Additional file 1: — Multilingual abstracts in the six official working languages of the United Nations. (PDF 809 kb) [file 40249_2017_248_MOESM1_ESM.pdf]

Translation of the abstract into the six official working languages of the United Nations

البلهارسيا المعوية في أوغندا في أماكن العلو المرتفع (< 1400 م): استطلاعات مسح لدراسة الرخويات ومسح وبائي في جبل إيلجون وفي بحيرات فوهة البركان فورت بورتال تكشف الحاجة إلى علاج كيميائي وقائي إضافي

ميشيل جيم ستانتون، موسيز أدريكو، موسى أرينايتوي، أليسون هويل، جولييت ديفيز، جيليان أليسون، إ. جيمس لاكورس، إدريداه موهيكي، نارسيس ب كاباترين، ج. روسل ستوزارد

ملخص

خلفية: البلهارسيا المعوية لها أهمية في الصحة العامة في أوغندا ولكن المجتمعات التي تعيش في ارتفاع يزيد على 1400 متر لا تقع ضمن الأماكن المستهدفة للسيطرة على المرض حيث يعتقد أنه من غير المرجح أن تكون ناقل طبيعي. لتقييم حدود الارتفاع والسكان المعرضين للخطر، أجريت مسوحات لدراسة الرخويات ومسوحات وبائية موحدة في جبل إيلجون (1139 م-3937 م)، وفي بحيرات فوهة البركان فورت بورتال وفي جبال روينزوري (1123 م-4050 م).

الأساليب: تم فحص سبعين من موائل المياه العذبة [جبل إيلجون (37)، بحيرات فوهة البركان فورت بورتال (23)، جبال روينزوري (8) بحيرة ألبرت (2)] بحثا عن أنواع البيومفلاريا. وسجلت درجة حرارة الماء، ودرجة الحموضة والموصلية. وأجري فحص طفيليات لـ 756 من تلاميذ المدارس [جبل إيلجون (300)، بحيرات فوهة البركان فورت بورتال (456)] بواسطة فحص البراز تحت المجهر لمسحات كاتو-كاتز مكررة من عينتي براز متتاليين بواسطة الفحوصات التشخيصية المستضد (غَميسَة البول-المستضد الكاثودي المنتشر) والأجسام المضادة (SEA-ELISA).

النتائج: قوقع بيومفيالريا. تم العثور على ما يصل إلى 1951 متر في جبل إيلجون و1567 م في بحيرات فوهة البركان فورت بورتال. على الرغم من عدم إخراج الحلزون من جبل ايلجون للسركاريا، يشير التحليل الجزيئي إلى أن 7.1٪ من عينات القواقع المأخوذة من ارتفاعات تزيد على 1400 م تحتوي على الحمض النووي للبلهارسيا المعوية. في بحيرات فوهة البركان فورت بورتال أخرجت ثلاثة من قواقع البلهارسيا السركاريا. تم قياس انتشار البلهارسيا المعوية في طلبة المدارس بواسطة أسلوب كاتو-كاتز (جبل إيلجون = 18.3٪ مقابل بحيرات فوهة البركان فورت بورتال = 34.4٪) وغميسَة البول-المستضد الكاثودي المنتشر (18.3٪ مقابل 34.4٪) وSEA-ELISA (42.3٪ مقابل 63.7٪) وهو ما يظهر وجود تداعيات سلبية مع زيادة الارتفاع مع بعض الأدلة على العدوى عند ارتفاعات تصل إلى 2000 م.

الاستنتاجات: خلافا للتوقعات، تبين هذه الاستطلاعات بوضوح أن الانتقال الطبيعي للبلهارسيا المعوية يحدث في الارتفاعات التي تزيد عن 1400 متر، ومن المحتمل أن يمتد حتى ارتفاع 2000 م. استخدام التنبؤات الوبائية المكانية يظهر أن هناك ستة ملايين شخص آخرين معرضين للخطر، وهذا يشير للحاجة إلى التوسع في العلاج الكيميائي الوقائي في أوغندا.

Translated from English version into Arabic by Mahmoud Sami, through

TRANSLATORS
WITHOUT BORDERS

乌干达高海拔地区的肠道血吸虫病（>1 400 m）：埃尔贡山和波特尔堡火山口湖的贝类学和流行病学调查显示需要额外的预防性化疗

Michelle C. Stanton, Moses Adriko, Moses Arinaitwe, Alison Howell, Juliet Davies, Gillian Allison, E. James LaCourse, Edridah Muheki, Narcis B. Kabatereine, J. Russell Stothard

摘要

引言：肠血吸虫病在乌干达具有重要的公共卫生意义，但未对对海拔超过 1 400 m 的社区进行防治，因为人们认为在那里该病不可能自然传播。为了评估海拔界限和高危人群，在埃尔贡山（1 139 m-3 937 m）、波特尔堡火山口湖和鲁文佐里山脉（1 123 m-4 050 m）开展了贝类学和流行病学的联合调查。
方法：对 70 个淡水栖息地[埃尔贡山（37 个）、波特尔堡火山口湖（23 个）、鲁文佐里山脉（8 个）、阿尔伯特湖（2 个）]进行双脐螺（*Biomphalaria*）种类调查。记录水的温度、pH 值和导电性。对 756 名学生[埃尔贡山（300 名）、波特尔堡火山口湖（456 名）]进行了抗原（尿-循环阴极抗原试纸，CCA urine-dipsticks）、抗体（SEA-ELISA）检测和 Kato-Katz 法进行粪检。
结果：在埃尔贡山海拔 1 951 m 和波特尔堡火山口湖海拔 1 567 m 处发现双脐螺。尽管埃尔贡山无钉螺逸出尾蚴，但分子检测结果显示，在海拔超过 1 400 m 采集到的钉螺样品有 7.1%检测到曼氏血吸虫 DNA；在波特尔堡火山口湖有 3 个钉螺逸出尾蚴。Kato-Katz 法、CCA urine-dipsticks 和 SEA-ELISA 的检验结果显示，埃尔贡山和波特尔堡火山口湖学生的肠道血吸虫病流行率分别为 18.3%、18.3%、42.3%和 34.4%、34.4%、63.7%，表明在海拔 2 000 m 内海拔增高与感染负相关。
结论：与期望相反的是，这些调查清楚地表明在超过海拔 1 400 m 时肠道血吸虫病可自然传播，有可能延伸至海拔 2 000 m。运用空间流行病学预测，该地区现有额外的六百万人群受到威胁，预示着在乌干达需要扩大预防性化疗。

Translated from English version into Chinese by Yin-Long Li, edited by Pin Yang

CHINESE CENTER FOR DISEASE CONTROL AND PREVENTION
NATIONAL INSTITUTE OF PARASITIC DISEASES

La schistosomiase intestinale dans les régions d'altitude (>1 400 m) en Uganda: des relevés malacologiques et épidémiologiques sur le mont Elgon et dans les lacs de cratère de Fort Portal révèlent de nouveaux besoins en chimiothérapie préventive

Michelle C. Stanton, Moses Adriko, Moses Arinaitwe, Alison Howell, Juliet Davies, Gillian Allison, E. James LaCourse, Edridah Muheki, Narcis B. Kabatereine, J. Russell Stothard

Résumé

Contexte: La schistosomiase intestinale est une préoccupation de santé publique en Uganda, mais les populations vivant au-dessus de 1 400 m ne sont pas contrôlées car la transmission naturelle est jugée peu probable à cette altitude. Afin d'évaluer les limites d'altitude et les populations à risque, des relevés malacologiques et épidémiologiques conjoints ont été réalisés sur le mont Elgon (1 139 m-3 937 m), dans les lacs de cratère de Fort Portal et dans les monts Rwenzori (1 123 m-4 050 m).
Méthodes: Soixante-dix habitats d'eau douce (mont Elgon [37], lacs de cratère de Fort Portal [23], monts Rwenzori [8] et lac Albert [2]) ont été inspectés à la recherche d'espèces du genre *Biomphalaria*. La température, le pH et la conductivité de l'eau ont été relevés. Un examen parasitologique de 756 écoliers (mont Elgon [300], lacs de cratère de Fort Portal [456]) par étude au microscope de doubles frottis fécaux Kato-Katz provenant de deux échantillons de selles consécutifs a été renforcé avec des essais de diagnostic des antigènes (bandelette réactive CCA dans l'urine) et les anticorps (ELISA pour la détection des SEA).

Résultats: Des espèces de *Biomphalaria* ont été retrouvées jusqu'à 1 951 m sur le mont Elgon et 1 567 m dans les lacs de cratère de Fort Portal. Bien qu'aucun escargot du mont Elgon n'ait excrété de cercaires, l'analyse moléculaire a indiqué que 7,1 % des mollusques prélevés au-dessus de 1 400 m portaient l'ADN de *Schistosoma mansoni* ; quand aux lacs de cratère de Fort Portal, trois escargots qui y avaient été prélevés excrétaient des cercaires de schistosome. La prévalence de la schistosomiase intestinale, mesurée sur des écoliers par la méthode Kato-Katz (mont Elgon = 18,3 % et lacs de cratère de Fort Portal = 34,4 %), bandelette réactive CCA dans les urines (18,3 % et 34,4 %) et SEA-ELISA (42,3 % et 63,7 %), fait apparaître des associations négatives avec l'altitude croissante, avec quelques indices d'infestation jusqu'à 2000 m.
Conclusions: Contrairement aux attentes, ces relevés montrent clairement que la transmission naturelle de la schistosomiase africaine est possible au-dessus de 1 400 m et peut-être jusqu'à 2000 m. Suivant les prédictions épidémiologiques spatiales, cela fait entrer quelque six millions de personnes dans la population à risque et nécessite une extension de la chimiothérapie préventive en Uganda.

Translated from English version into French by Suzanne Assenat, through

TRANSLATORS
WITHOUT BORDERS

Кишечный шистосомоз на большой высоте (> 1 400 м) в Уганде: малакологического и эпидемиологические исследования на горе Элгон и в кратерных озерах Форт-Портала показывают дополнительные профилактические потребности химиотерапии

Мишель С. Шантон, Моисей Адрико, Моисей Аринаитве, Алисон Хоуэль, Джульет Дэвис, Джиллиан Эллисон, Е. Джеймс ЛаКорс, Эдридан Мухеки, Нарцисс Б. Кабатерайн, Дж. Руссел Стотхард

Аннотация

Предпосылки и цель исследования: Кишечный шистосомоз имеет важное значение для общественного здравоохранения в Уганде, но для общин, живущих на местах выше чем 1 400 м над уровнем моря контроль не предосмотрены, потому что, естественная передача болезни считается маловероятным. Для оценки границ высоты и населения в находящиеся в зоне риска, слитные малакологические и эпидемиологические обследования были проведены на горе Элгон (1 139 м-3 937 м), в кратерных озерах Форт-Портала и в Рувензори (1 123 м-4 050 м).
Методы: Семьдесят пресноводных сред обитания [Гора Элгон (37), кратерная озера Форт-Портал (23), Рувензори (8) и озеро Альберт (2)] были проверены на предмет видов Biomphalaria. Температура воды, pH и проводимость отмечены. Паразитологическое обследование 756 школьников [Гора Элгон (300), кратерная озера Форт-Портал (456)] фекальное микроскопие мазков дублирующих Като-Katz из двух последовательных проб фекалий была подкреплена диагностическими анализами антиген (моча-CCA щупом) и антитело (море- ELISA).

Результаты: Biomphalaria SPP. был найден на местах выше до 1 951 м над уровнем моря на горе Элгон и выше до 1 567 мн ад уровнем моря в кратерных озерах Форт-Портал. Хотя ни одна улитка выделил с горы Элгон церкарии, молекулярный анализ оценивается на 7,1% по уликам, отобранных на высоте более 1 400 м, как имеющие ДНК Шистосомы Мансони; в кратерных озерах Форт-Портала три улиток выделил шистосом церкарии. Распространенность кишечной шистосомоз как измерено у детей школьного возраста Като-Кац (на горе Элгон = 18,3% в сопоставлении с кратерной озерой Форт-Портал = 34,4%), мочи щупы ЦКА (18,3% в сопоставлении с 34,4%) и SEA-ELISA (42,3% в сопоставлении с. 63,7%) показали негативные ассоциации с увеличением высоты с некоторыми признаками инфекции до 2 000 м.
Выводы: Вопреки ожиданиям, эти исследования ясно показывают, что естественная передача кишечного шистосомоза происходит на местах выше чем 1 400 м над уровнем моря, возможно расширение до 2 000 м. Использование пространственных эпидемиологических прогнозов, теперь эти местности ставить в опасность некоторых дополнительных шесть миллионов человек, и это свидетельствует о расширении профилактических потребностей химиотерапии в Уганде.

Translated from English version into Russian by Turdimurot Rakhmonov, through

TRANSLATORS
WITHOUT BORDERS

Esquistosomiasis intestinal en uganda a gran altitud (> 1 400 m): Estudios malacológicos y epidemiológicos en el Monte Elgon y en los lagos del cráter de Fort Portal revelan necesidades adicionales de quimioterapia preventiva.

Michelle C. Stanton, Moses Adriko, Moses Arinaitwe, Alison Howell, Juliet Davies, Gillian Allison, E. James LaCourse, Edridah Muheki, Narcis B. Kabatereine, J. Russell Stothard

Resumen

Antecedentes: La esquistosomiasis intestinal es de pública importancia en Uganda, pero las comunidades que se encuentran sobre los 1 400 m de altitud no son objetivo de tratamiento para control ya que la transmisión natural es considerada poco probable. Para evaluar las fronteras de la altitud y el riesgo de las poblaciones, se realizaron estudios epidemiológicos y malacológicos conjuntos en el Monte Elgon (1 139 m – 3 937 m), en los lagos del cráter de Fort Portal y en las montañas Rwenzori (1 123 m – 4 050 m).
Métodos: Se examinaron setenta hábitats de agua dulce [Monte Elgon (37), lagos del cráter de Fort Portal (23), Montañas Rwenzori (8) y Lago Albert (2)] para especies de *Biomphalaria*. Se registró la temperatura del agua, el pH y la conductividad.
Un examen parasitológico de 756 escolares [Monte Elgon (300), lagos del cráter de Fort Portal (456)] por microscopía fecal de frotis de Kato-Katz duplicados, de dos muestras de heces consecutivas, fue reforzado con antígeno (tiras reactivas CCA de orina) y pruebas diagnósticas de anticuerpo (SEA-ELISA).

Resultados: Se encontró *Biomphalaria* spp. hasta 1 951 m en el Monte Elgon y 1 567 m en los lagos del cráter de Fort Portal. Aunque ningún caracol del Monte Elgon arrojó cercariae, el análisis molecular estimó que el 7,1% de los caracoles fueron muestreados a altitudes superiores a 1 400 m con ADN de *Schistosoma mansoni*; En los lagos del cráter de Fort Portal tres caracoles arrojaron cercariae de esquistosoma. La prevalencia de la esquistosomiasis intestinal medida en escolares por Kato-Katz (Monte Elgon = 18,3% vs. lagos del cráter de Fort Portal = 34,4%), tiras reactivas-CCA de orina (18,3% vs. 34,4%) y SEA-ELISA (42,3% vs. 63,7%), mostraron asociaciones negativas con altitud creciente, con alguna evidencia de infección hasta los 2 000 m.
Conclusiones: Contrariamente a lo que se esperaba, estos estudios muestran claramente que la transmisión natural de la esquistosomiasis intestinal ocurre a los 1 400 m y posiblemente hasta los 2 000 m. Usando predicciones epidemiológicas espaciales, esto coloca en riesgo a más de seis millones de personas, lo que denota una necesidad de aumento de la quimioterapia preventiva en Uganda.

Translated from English version into Spanish by patriciacassoni, through

TRANSLATORS
WITHOUT BORDERS
